# SAMPLEX: Automatic mapping of perturbed and unperturbed regions of proteins and complexes

**DOI:** 10.1186/1471-2105-11-51

**Published:** 2010-01-26

**Authors:** Mickaël Krzeminski, Karine Loth, Rolf Boelens, Alexandre MJJ Bonvin

**Affiliations:** 1 Bijvoet Center for Biomolecular Research, Science Faculty, Utrecht University, 3584 CH, Utrecht, The Netherlands; 2 Centre de biophysique moléculaire, UPR n°4301 CNRS, affiliated to the University of Orléans and to Inserm, rue Charles Sadron, 45071 Orléans Cedex 2, France

## Abstract

**Background:**

The activity of proteins within the cell is characterized by their motions, flexibility, interactions or even the particularly intriguing case of partially unfolded states. In the last two cases, a part of the protein is affected either by binding or unfolding and the detection of the respective perturbed and unperturbed region(s) is a fundamental part of the structural characterization of these states. This can be achieved by comparing experimental data of the same protein in two different states (bound/unbound, folded/unfolded). For instance, measurements of chemical shift perturbations (CSPs) from NMR ^1^H-^15^N HSQC experiments gives an excellent opportunity to discriminate both moieties.

**Results:**

We describe an innovative, automatic and unbiased method to distinguish perturbed and unperturbed regions in a protein existing in two distinct states (folded/partially unfolded, bound/unbound). The SAMPLEX program takes as input a set of data and the corresponding three-dimensional structure and returns the confidence for each residue to be in a perturbed or unperturbed state. Its performance is demonstrated for different applications including the prediction of disordered regions in partially unfolded proteins and of interacting regions in protein complexes.

**Conclusions:**

The proposed approach is suitable for partially unfolded states of proteins, local perturbations due to small ligands and protein-protein interfaces. The method is not restricted to NMR data, but is generic and can be applied to a wide variety of information.

## Background

During the last decennia, there has been a growing interest in biological system dynamics, which consist for instance of interactions between proteins and folding pathways. This also includes proteins that partially unfold under specific environmental stimuli (taxis), and act as intermediates in a cascade of events [1]. In all cases, only a part of the protein is involved in the biological process while the remaining part stays dormant. Indeed, in a protein-protein complex, only the interaction surface is often affected, even though, sometimes, a larger part of the protein is modified, like in the case of allosteric interactions or conformational changes upon binding. As for partially unfolded proteins, which are typically composed of mobile regions linked to a folded core, they show some flexibility due to the internal motions of the protein and the reorganization of the unfolded part upon partial unfolding. In order to structurally characterize such systems, it is crucial to distinguish the unperturbed regions from the perturbed ones.

Nuclear Magnetic Resonance (NMR) is particularly convenient for monitoring, at atomic level, structural and/or environmental changes, which occur upon binding to a partner molecule or at (partial) unfolding. In particular, heteronuclear shift correlation experiments, such as ^1^H-^15^N HSQC, are very useful to detect alterations in the electronic environment of atoms which affect their chemical shifts. Hence, comparison of HSQC spectra of a molecule in two different states (bound/unbound, folded/unfolded) allows the detection of affected residues by analyzing the chemical shift perturbations (CSPs) [2-4].

A common method to distinguish unperturbed residues from others is to reject the ones for which the CSP is higher than a pre-defined threshold that usually corresponds to the average of all CSPs plus one or two times their standard deviation. This procedure is repeated with the remaining residues, until no rejection occurs anymore. The choice of a cut-off can be biased by subjectivity in the selection process. In addition, there is no simple relationship between the amount of chemical shift perturbations and the magnitude of the perturbation; this sometimes results in biased data (like very low CSP for a residue that is flanked by two others with very high CSPs), leading to misinterpretations. With that respect, it might be more relevant to consider not only the CSP of the concerned residue or its sequential neighbors, but also the CSPs of the residues close to it in the 3D structure.

This problem has previously been addressed by Kalbitzer et al. [5] for the case of protein-protein interactions. Their method yields the probability for a given residue to be involved in the interaction by comparing its CSP and the average CSP of the same residue found in all complexes of the BMRB database [6]. This approach however depends on the way the CSP is calculated [5] and an user-defined cut-off. In the present study we present an automated and less biased procedure to discriminate perturbed regions from unperturbed ones in a protein using its three dimensional structure and a set of experimental data. We will use in this work CSP data, but the method is generic and can be used for other types of data as well. The program we developed for this purpose, SAMPLEX (Smoothed Automatic Mapping of Protein from Listed Extremes), is based on a topologic approach and can overcome problems due to peak overlap or to sparse data. We tested our method on several systems, including protein complexes and partially unfolded proteins, for which NMR data were available.

## Implementation

Considering a system existing in two different states, the CSP of the atoms of a given residue can be calculated from the HSQC spectra of the protein obtained for each state as follows [2]:(1)

where *ΔH*_*N*_, *ΔN*, *ΔC*_*α *_and *ΔC*_*O *_are the chemical shift differences of the hydrogen (H_N_), the amide nitrogen (N), the alpha carbon (C_α_) and the carbon of the carboxyl group (C_O_) of the backbone, respectively, and *SW*_*N*_, *SW*_*Cα *_and *SW*_*CO *_are the spectral width ratio between H_N _and N, H_N _and C_α _and H_N _and C_O_, respectively [3].

From these CSPs determined for each residue (ideally) and the known native structure of the protein of interest, SAMPLEX automatically delimits the perturbed regions of the protein. The method has been divided into four consecutive steps that aim at defining groups of residues that belong to a similar state (perturbed/unperturbed) within the protein.

### Step 1. Attribution of confidences

In an ensemble of CSPs values, a way to reflect how significantly different is a CSP value compared to all others is to calculate the factor *k *defined as:(2)

where *CSP*_*i *_is the CSP of the residue *i*, *μ *the average of all CSPs and *σ *the standard deviation. The factor *k *can be positive or negative. We subsequently transform these factors into values *ρ*_*i *_such that the highest CSP value(s) becomes 1, the lowest -1 and all others have intermediate values. From now on, we will call *ρ*_*i *_the confidence of the residue *i*. However, to avoid that the highest and lowest CSP values, which can be extreme, dominate this transformation, we first determine minimum factors *k*_*high *_and *k*_*low*_. For this, we run several times (roughly 1000 times the number of available data) the following process:

• Selection of a sub-ensemble made of the highest, the lowest and randomly selected CSP values such that the size of this sub-ensemble corresponds to 15% of the number of available data.

• Calculation of the factor *k *for this sub-ensemble using the equation 1.

We then define *k*_*high *_and *k*_*low *_as the lowest and highest *k *values found among all runs performed.

Then, to determine the confidence *ρ*_*i *_of each residue *i*, we perform the following steps:

1. Initialization of the variable *α*_*ι *_= 0.

2. Selection of a sub-ensemble made of the CSP value of residue *i *and randomly selected CSP values such that the size of this sub-ensemble corresponds to 15% of the number of available data.

3. Calculation of the factor *k*_*i *_for this given ensemble.

4. Increment *α*_*i *_by Γ(*k*_*i*_) defined as:(3)

The value of 0 accounts for a null standard deviation, which is not informative.

5. Steps 2 to 4 are repeated *n_trial *= 100 times the number of available data.

6. The confidence *ρ*_*i *_of residue *i *is finally given by the quotient of *α*_*i *_over *n_trial*.

### Step 2. Inference of missing residues

In some cases, no data are available for a given residue (e.g. in the case of CSPs, due to missing assignments, line broadening, prolines...) To overcome such lack of information, we can still infer a confidence by the mean of multivariate interpolation, using an inverse distance weighting approach: a delusive confidence *ρ*_*i *_is assigned based on the residue direct environment using a barycentric method which depends on the distances to its neighbors as follows:(4)

where *i *is the residue to infer, *j *a neighbor of *i *within a cut-off distance of 7.5 Å, *n *the total number of neighbors and *φ*_*ij *_a sigmoidal function. This latter depends on the distance between the non-weighted barycentres of atoms of residues i to residue j for which we have some data; it is defined so that its value is very close to 1 when the distance is null and very close to 0 when the distance is equal to the predefined cut-off of 7.5 Å:(5)

where *d*_*i*__*j *_is the distance between residues i and j, *c *the predefined cut-off, *ε *is a very small number set to 10^-3 ^in this work, *α*_*ε *_a constant equal to *ε*/(1-*ε*) and *λ( c) *a function defined as follows:(6)

At least two neighboring residues with available experimental data should be detected otherwise the residue is excluded from further calculations and no decision about its state is made in the end. When an ensemble of structures is provided, neighbors are defined by considering the average distance over all models. This allows taking into account possible conformational heterogeneity into the procedure.

### Step 3. Homogenization of confidences

The confidence *ρ*_*i *_of each residue is then adjusted based on its neighborhood in the 3D structure of the protein. We apply an iterative process until the root mean square deviation between two consecutive steps is becoming lower than a threshold value of 10^-5^. This results in the creation of blocks of residues for which the homogenized confidences are close to each other. Each step of this process consists of attributing a new confidence  to residue *i *as follows:(7)

In this formula,  is the newly attributed confidence of residue *i*,  the starting confidence, *n *the number of neighbors of residue *i*, and *φ*_*ij *_the sigmoidal function defined in Eq. 5.

### Step 4. Final decision

From the homogenized confidences, we define all residues with a confidence higher than 0.05 as being in a perturbed state and all residues with a confidence lower than -0.05 as remaining in their unperturbed state. The residues in between those two values are considered to be in an ambiguous state, which can mean that they might be close to a perturbed region or indirectly affected by a perturbed region.

## Results and Discussion

The selection approach described above was tested on five distinct systems with different characteristics. The various test cases are representative of both binding - and partial unfolding - induced CSP data. In the case of protein-protein complexes, the situation is delicate in the sense that the measure of the accuracy of the selection depends on the way we define the interface and on the quality of the structures. In this work, we defined the residues at the interface as the ones having at least one atom of the backbone within 5 Å of any atom of the other subunit, excluding hydrogen atoms. In the case of an ensemble of conformations, we consider that a contact is effective if it is found in at least one model. Graphical results are given for each test case [Additional file 1].

### Performance of diverse test cases

#### Chymotrypsin inhibitor 2 *(CI2) *subunit in complex with the Subtilisin BPN'

**Figure 1:**
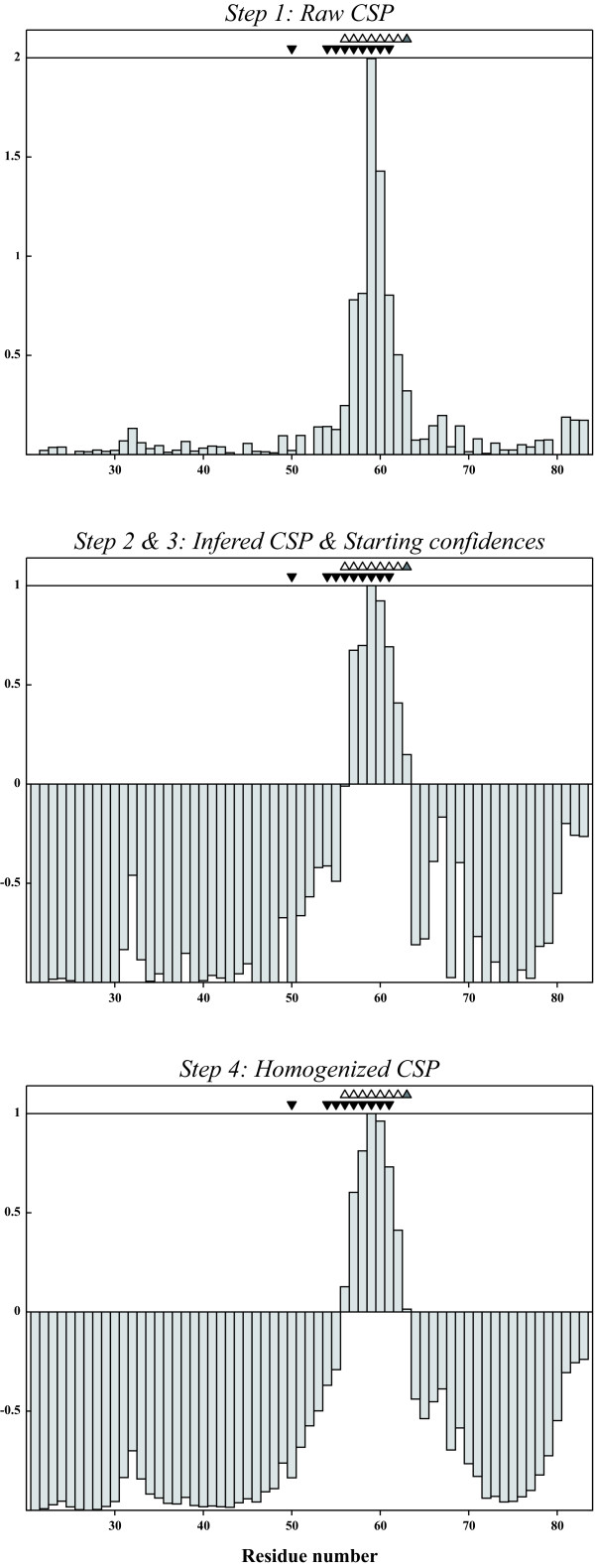
**Chemical shift perturbations and derived confidences as function of the residue sequence for CI2 (unbound and bound to BPN')**. Top: Raw CSP data; Middle: Starting confidences; Bottom: Confidences after homogenization. The triangles filled in black show residues involved in the interaction between CI-2 and BPN', the ones filled in grey show the selection made by SAMPLEX and the open ones, residues in an intermediate state.

Subtilisin BPN' is a serine protease [7] which can be inhibited by the chymotrypsin inhibitor 2, an 83 amino acid protein with a disordered N-terminal tail [8]. Figure 1 shows the evolution of the residue selection for the CI2 subunit of the Subtilisin/CI2 complex as a function of the raw CSP data (C. van Heijnoort and L. Koharudin, personal communication), starting confidences and the resulting homogenized confidences. In this case we took for the selection process the structures from the complex (PDB entry 1LW6) [8].

**Figure 2:**
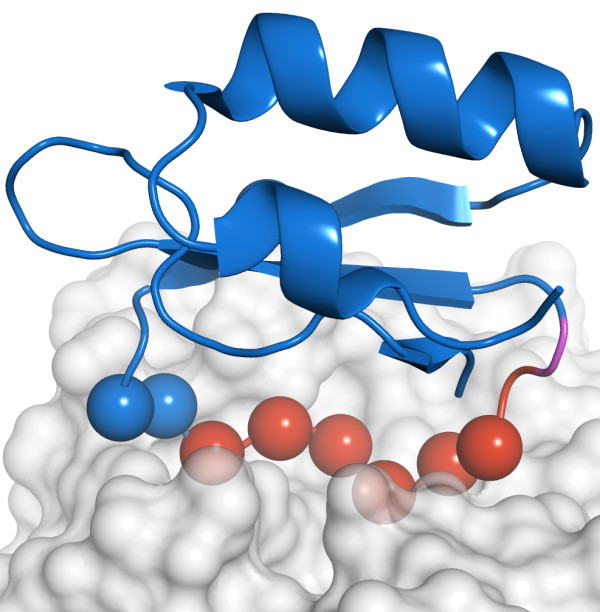
**Selection of perturbed regions of CI2 after complexation with BPN'**. In red and purple are displayed residues selected by SAMPLEX in a non-native like environment and in an ambiguous state, respectively. Spheres represent the interface based on the criterion defined in this paper: a residue belongs to the interface if at least one atom of the backbone is at a maximum distance of 5Å of any atom of the other subunit, excluding hydrogen atoms.

For CI2, SAMPLEX estimates that residues 56-62 are perturbed and residue 63 is in an ambiguous state. The CI2/BPN' X-ray complex, solved at 1.50 Å (PDB entry 1LW6) [8], shows that residues 50 and 54 to 61 are involved in the interaction (Figure 2). Residue 50 however is only defined as interacting based on the proximity of its carbonyl oxygen to a side chain oxygen of D^99 ^in BPN' (distance between both oxygens is 4.8 Å). This can explain why its amide group is not affected by the binding and therefore not selected by SAMPLEX.

For BPN', the situation is more complicated because of the size of the protein (281 residues) and the lack of data (16% of confidences had to be inferred). Residues 33, 97, 99-109, 126-128, 141, 154-156, 167-171 and 218-219 are predicted by SAMPLEX as being in a non-native-like environment, and residues 65, 98 and 220 as being in an ambiguous state. The interface measured from the X-ray complex includes residues 99-104, 125-128, 154-157, 167, 218-221. Besides residue 141, all residues selected by SAMPLEX are either in or close to the interface (see figure 2) and consequently could be perturbed due to the interaction with CI2. Residue 141, which is not close to the interface, shows the second highest experimental CSP; this could be due to an indirect perturbation (e.g. conformational change).

#### The Ubiquitin-conjugating enzyme E2-7 (UBCH5) in complex with CCR4-NOT (CNOT4)

UBCH5 is a human protein involved in the ubiquitination of proteins [9]. It interacts with CNOT4, a transcription factor [10]. From the unbound form of the protein (PDB entry: 2ESO) [11], our algorithm estimates that residues 1-12, 16-17, 60-64, 88 and 94-101 of UBCH5 are perturbed. No Residue is classified in an ambiguous state. The contacts defined from the structural model obtained with HADDOCK [12,13] (PDB entry: 1UR6) 8-59, 61-63, 88 and 91 to 96 are involved in the interaction. Although this structural model was obtained based on chemical shift information, we are confident that it presents an accurate picture of the complex due to its high homology to the UbcH7-c-Cbl complex solved by X-ray crystallography (PDB entry 1FBV) [14].

**Figure 3:**
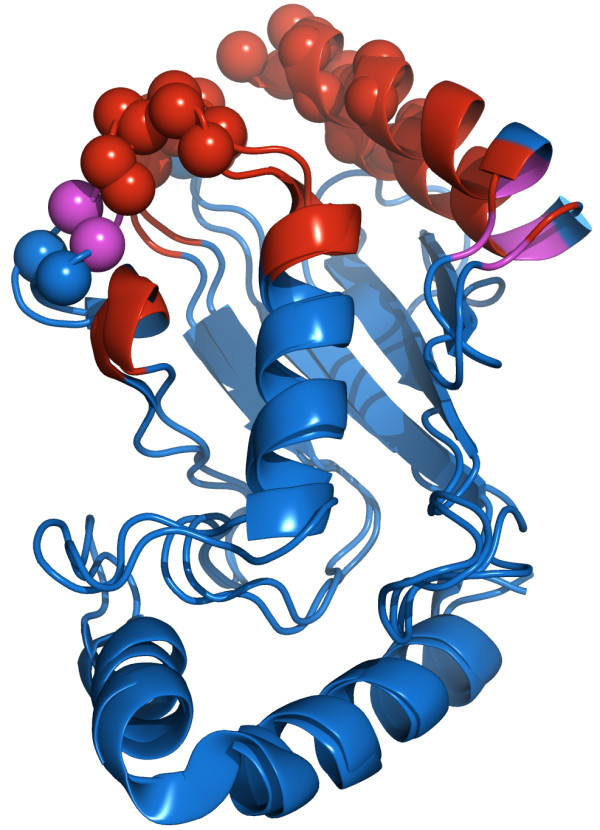
**Alignment of the free and the bound form of UBCH5**. In red are depicted perturbed residues as predicted by SAMPLEX, in purple are residues in an intermediate state. The real interface defined from 1UR6 is displayed with spheres. We clearly see the shift of the first helix and of residues 87-88 (black arrow) upon the formation of the complex.

SAMPLEX finds the correct regions, but extends the one of the N-terminal helix (residues 1-13, 16-17). When we align the structures of the unbound and bound forms of UBCH5, ignoring this first helix (Figure 3), we clearly see that the position of this helix has shifted. In the same way, we also notice a shift of residues 87 and 88. The region delimited by the residues 93 to 101 corresponds to a loop that is in contact with CNOT4. Hence, these regions might be perturbed accounting for the selection made by SAMPLEX.

On the CNOT4 side, the predictions turn out to be rather accurate. The algorithm predicts residues 15 to 19, 41, 45, 47 to 50 and 53 to 57 as perturbed and residues 14, 40, 46 and 52 in an intermediate state. The interface from the structural model comprises residues 15 to 20, 40 to 41, 44 to 45, 48 to 50, 52 to 55 and 57.

#### Colicin E9 domain (CE9) subunit in complex with its cognate immunity protein (IM9)

CE9 is a small bacterial protein with cytotoxic endonuclease activity. The structures of its unbound and bound forms have been solved by X-ray at 1.80 Å and 1.70 Å, respectively (PDB entries 1FSJ and 1EMW, respectively) [15]. In addition, NMR chemical shifts have been measured for both the unbound and bound forms (BMRB accession numbers 4352 and 4293, respectively) [16,17]. As for its partner, Im9, both bound (1EMW) and unbound forms are also available. The latter has been solved by NMR (PDB code 1IMP) [18]. Chemical shifts are available for both forms (BMRB accession numbers 4115 and 4116) [19].

For E9, the perturbed region estimated by SAMPLEX encloses residues 23, 72-79, 86-87 and 94-99. Residues 70, 80, 85 and 93 are in an ambiguous state. From the crystal structure, residues 72-75, 77-, 81, 83, 86, 89 and 97 are at the interface. In the case of Im9, SAMPLEX defines residues 24, 30-37, 47-57 and 82 as perturbed and residue 38 as ambiguous. The crystal structure indicates that residues 23-25, 30, 33-34, 50-51, 54-55 and 62 are involved in the interaction. SAMPLEX predicted a somewhat larger region in both proteins that correctly encompass the true interface. The additional predictions are mainly located in loops that can undergo conformational changes upon binding. Indeed, chemical shift perturbations alone cannot distinguish between direct interactions and induced conformational changes.

#### The lactose operon repressor and its inducer

The Lac repressor is a tetrameric protein that binds a specific operator on DNA and therefore inactivates the transcription of the enzymes involved in the metabolism of the lactose [20,21]. When a molecule of isopropyl-*β*-D-thiogalactoside (IPTG) is embedded in the repressor, this later is inhibited due to a structural rearrangement.

In this case, SAMPLEX predicts residues 66-82, 97, 124, 126-128, 133, 160-163, 192-197, 222, 276-280, 292-293, 319-320 and 322 as perturbed. In addition, residues 83, 95-96, 98-99, 125, 132, 221, 294 and 318 are classified as ambiguous state. The prediction corresponds to the binding pocket of IPTG and the beta sheet involved in the interaction between two monomers, which has previously been demonstrated as being perturbed [22].

#### The Photoactive Yellow Protein (PYP)

PYP is a 14 kDa protein found in the bacterium *Halorhodospira halophila*. Upon illumination at 446 nm, this protein partially unfolds and triggers the signal, which results in the movement by the bacterium away from the light [23-25]. The protein contains a PAS domain as found in many signaling proteins [26]. The chromophore is a p-coumaric acid, which undergoes a trans-to-cis isomerization after activation of the ground state. This isomerization results in the partial unfolding of the protein. A truncated form of PYP, Δ25-PYP, with the 25 first N-terminal amino acids deleted, shows a longer lifetime in the excited state (about 10 minutes), which allowed to record ^1^H-^15^N HSQC spectra and compare the ground and excited states [27].

Using as input the NMR structure of Δ25-PYP (PDB entry 1XFN) [27] the program defines the regions that comprise residues 29, 42-52, 54-58, 63-65, 67-74, 76-77 and 97 to 102 as perturbed, i.e., in this case, as partially unfolded. Residues 53, 66, 78-79 and 103 are in an ambiguous state. These regions contain the loop, which bears the chromophore (residue 69), residue E^46^, which is part of helix 3 and yields a proton to the chromophore before unfolding of the protein, and the loop flanked by beta sheets 4 and 5. This loop can be seen as the lead that covers the chromophore. Finally, a part of the helix 5 is perturbed, while all beta sheets stay in their native like environment. In conclusion, only the part surrounding the chromophore is predicted as affected.

Since the structure of the partially unfolded state of Δ25-PYP has been solved by NMR, we investigated the dependence of the predictions on the structure used: when running SAMPLEX from the unfolded structure (PDB code 1XFQ) [27] residues 28-29, 43-58, 63-65, 67-68, 70-74, 77-78, 97-102 are predicted as perturbed and residues 42, 66, 69, 76, 79, 103 as ambiguous. These results are quite similar (97% overlap considering perturbed and ambiguous regions together) to the ones obtained using the folded protein indicating that our method is robust with respect to the structure used.

#### An unperturbed protein

SAMPLEX can also assess whether data are indicative of a perturbation or not, by making use of the relative standard deviation *σ*_*R *_defined as *σ*_*R *_= *σ*/*μ*, where *μ *and *σ *are the mean and the standard deviation of all available data, respectively. Below a value of 25%, the program will consider that no part of the protein has been affected and will ask the user whether he wishes to continue. We tested this by analyzing HSQC spectra of the Lac Headpiece repressor in complex with DNA at two different frequencies (500 MHz and 900 MHz) and calculating CSP values from their difference. The resulting *σ*_*R *_was 5.9% and consequently SAMPLEX did not make any prediction. In the case of the other examples discussed in this paper, *σ*_*R *_increased to 200.7%, 286.4%, 141.6%, 115.5%, 153.0%, 200.8%, 117.6% and 88.9% for CI2, BPN', UBCH5, CNOT4, CE9, Im9, the lactose operon repressor and PYP, respectively.

### Comparison with general methods

Common methods that are often used to define perturbed regions within a protein from CSPs are:

i. to consider as perturbed all residues with a CSP value higher than the average of all available data plus one or two times their standard deviation

ii. to iterate a process in which residues with a CSP value higher than the average plus one or two times their standard deviation are rejected and start again with the remaining residues until no rejection occurs; all rejected are finally classified as perturbed.

We compared the performance of SAMPLEX with these two methods in the case of the complexes described above. The accuracy of the selection was measured by the Matthews coefficient correlation as described by Baldi *et al*. [28]:(8)

where *TP*, *FP*, *TN *and *FN *are the true positive (correctly predicted), false positive (wrongly predicted), true negative (correctly not predicted) and false negative (wrongly not predicted) residues, respectively. This coefficient reaches 1 when the prediction is perfect.

Table 1 summarizes the quality of the prediction for CI2, BPN' and UBCH5 using the various selection methods. We clearly see that SAMPLEX always outperforms the other two methods.

**Table 1:** Performance of SAMPLEX

Subunit	CI2	BPN'	UBCH5	CNOT4	CE9	Im9	Average
**SAMPLEX**	0.72	0.62	0.51	0.76	0.46	0.47	0.59
**Method A^1^**	0.72	0.41	0.20	0.46	0.23	0.20	0.37
**Method B^2^**	0.51	0.43	0.22	0.56	0.25	0.42	0.40

Comparison of the quality of the predictions for the interfaces of CI2, BPN', UBCH5, CNOT4, CE9 and IM9 using SAMPLEX and two commonly used methods as measured by the Matthews coefficient correlation (Eq. 8)

(1) Residues that have a CSP value higher than the average of all available CSP values plus one time their standard deviation are considered as perturbed

(2) Considering an iterative process, which consists on rejecting all residues with a CSP value higher than the average of all CSP values plus two times the standard deviation and starting again with remaining residues until no rejection occurs, all rejected residues are considered as perturbed.

## Conclusion

In this paper we have described a new un-biased strategy to distinguish perturbed from unperturbed regions in NMR spectra that define two different states of a protein. The program developed for this purpose, SAMPLEX, requires on the one hand the chemical shifts (or some other kind of data distinguishing the various states) of the protein in both the ground/free and the excited/bound state and on the other hand the structure (or an ensemble of structures) of the ground/free form as input. The method can be used to find the partially unfolded moiety of a protein, or in the case of proteins complexes, to define the interaction surface.

In this work, we only used CSP data from ^1^H-^15^N HSQC spectra and showed that they are already sufficient for a successful selection. However, since the result will depend on the quality and the amount of data, SAMPLEX would yield more accurate solution by including additional chemical shifts (^13^C_α_, ^13^C_O_...), in particular in the case of large proteins. It is worth noting again that, in the case of complexes, chemical shifts can report on both direct interaction and indirect effects such as remote conformational changes; results should thus always be carefully analyzed.

It should be noted that our selection method is generic and therefore not restricted to NMR CSP data. It can also be applied to e.g. order parameters from NMR relaxation data, protection factors calculated from H/D exchange experiments or any other experimental data as long as it provides information on a molecule at an atomic level. This makes it applicable to a large variety of problems in which some selection/classification needs to be performed.

## Software availability

SAMPLEX is written in python and is available free of charge. It can be downloaded from: http://www.nmr.chem.uu.nl/Software/samplex.

## Authors' contributions

MK and AMJJB developed the original idea and algorithm of SAMPLEX, programmed the method and prepared the first draft of the manuscript. AMJJB supervised and coordinated the entire project. KL and RB brought precious help to adjust some aspects of the original method due to the subtleties of interpretation of NMR data. All authors read and approved the final manuscript.

## Supplementary Material

Additional file 1 **Figures presenting the chemical shift perturbations and derived confidences as function of the residue sequence for all test cases discussed in the text**. Each column corresponds to one test case. Top: Raw CSP data; Middle: Starting confidences; Bottom: Confidences after homogenization. The horizontal red lines displayed on the graphic after homogenization delimit the perturbed (above the upper line), the unperturbed (bellow the lower line) and the intermediate (between the two lines) regions, as determined by SAMPLEX. Click here for file
