# Supplementary material for: SAMPLEX: Automatic mapping of perturbed and unperturbed regions of proteins and complexes
Source: BMC Bioinformatics. 2010 Jan 26;11:51. doi: 10.1186/1471-2105-11-51 (PMC2823710; doi:10.1186/1471-2105-11-51)

Supplementary material

SAMPLEX: Automatic mapping of perturbed and unperturbed regions of proteins and complexes.

[1]Mickaël Krzeminski, [1,2]Karine Loth, [1]Rolf Boelens and [1]Alexandre M.J.J. Bonvin*

[1]Bijvoet Center for Biomolecular Research, Science Faculty, Utrecht University, 3584 CH, Utrecht, The Netherlands
[2]Current address : Centre de biophysique moléculaire, UPR n°4301 CNRS, affiliated to the University of Orléans and to Inserm, rue Charles Sadron, 45071 Orléans Cedex 2, France

* Corresponding author

Email addresses:

MK: a.m.j.j.bonvin@uu.nl
KL: kloth@cnrs-orleans.fr
RB: r.boelens@uu.nl
AMJJB: a.m.j.j.bonvin@uu.nl

This document contains the chemical shift perturbations and derived confidences as function of the residue sequence for the diverse test cases described in the paper.

For each vertical panel, the top row displays raw CSP data, the middle row represents the starting and inferred confidences and the bottom row shows the confidences after homogenization, as well as the limits -0.05 and 0.05 (red bars) between which data are classified as ambiguous.

CI2 / Subtilisin

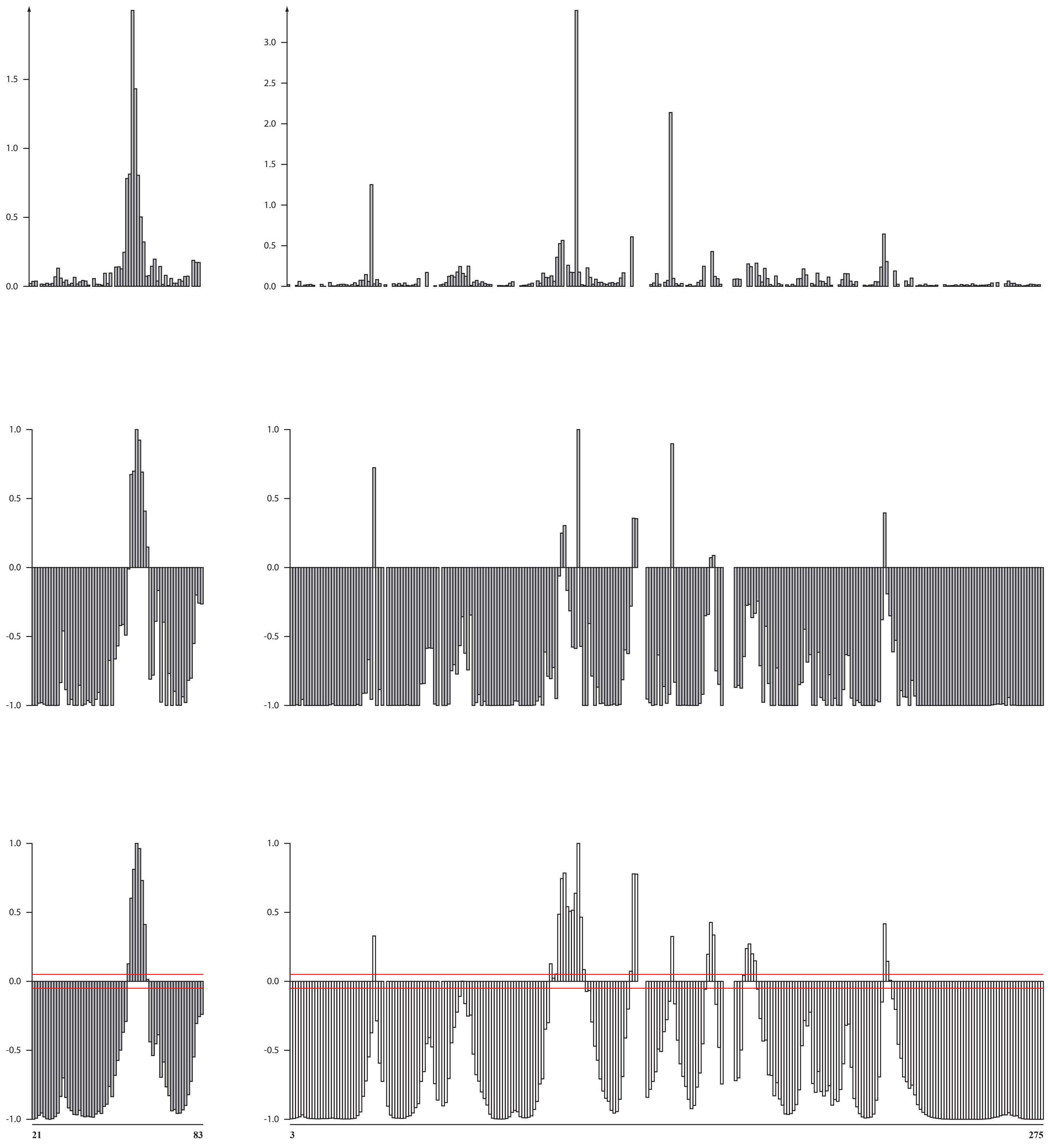

UBCH5 / CNOT4

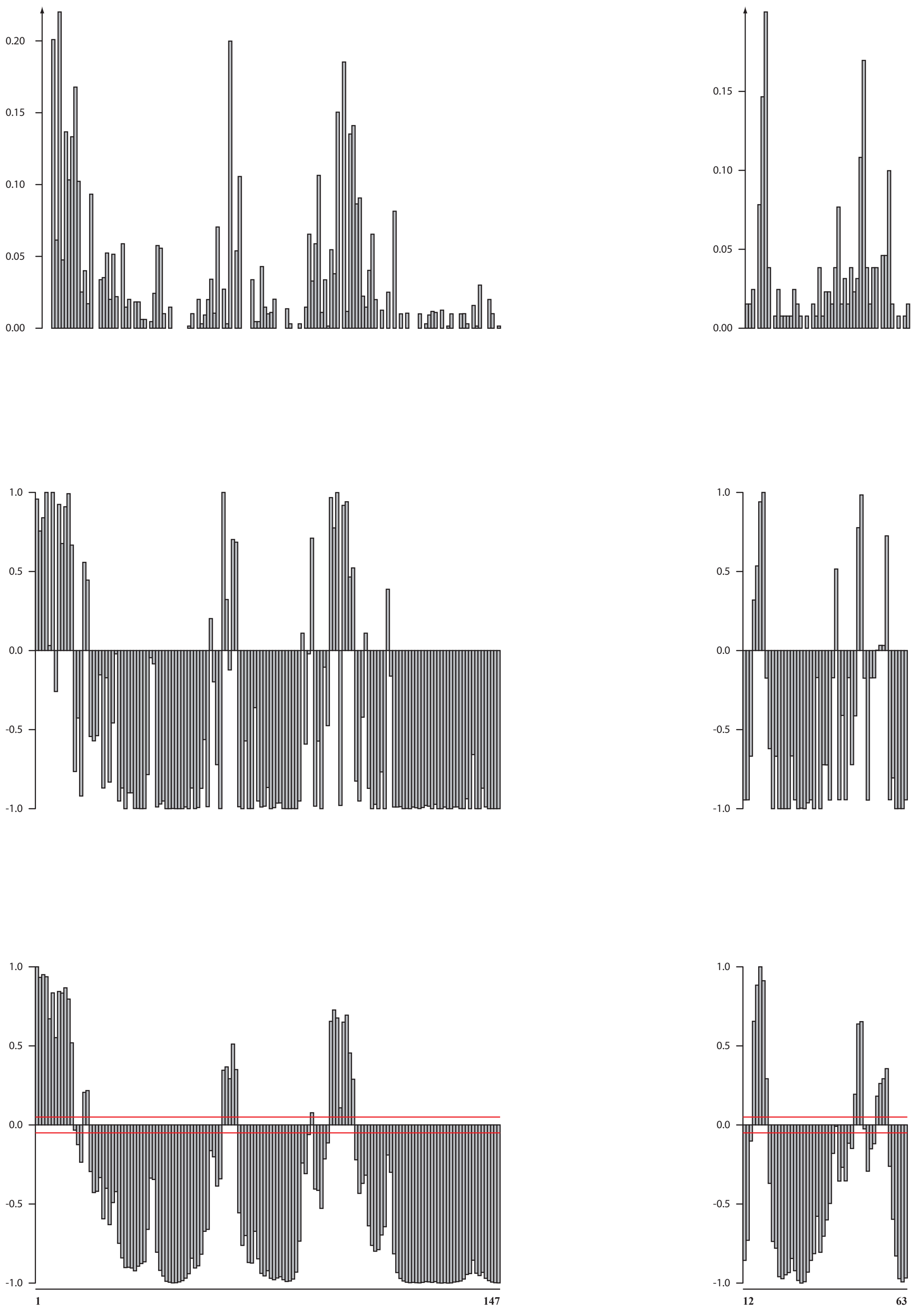

CE9 / Im9

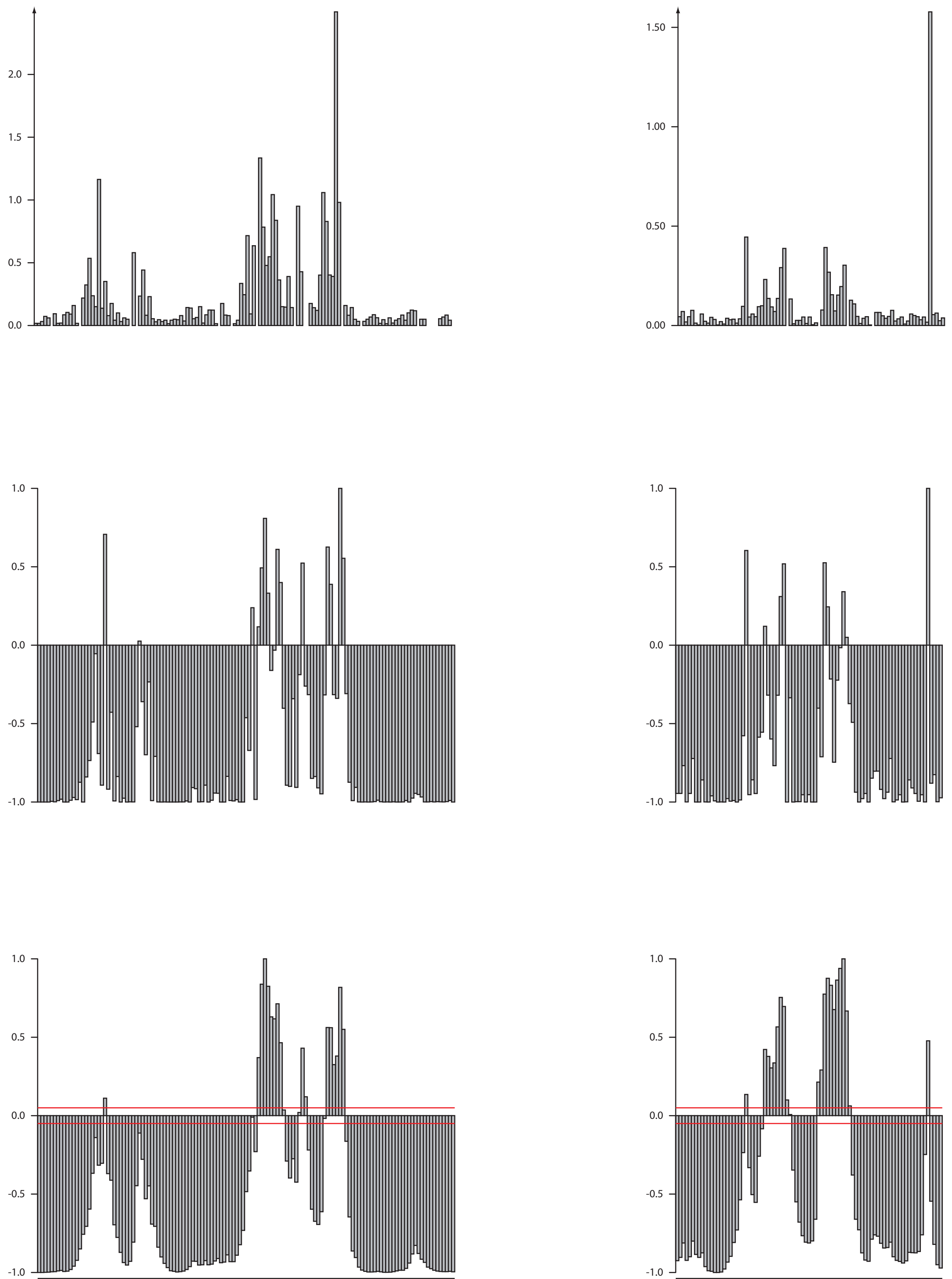

PYP **Lac repressor (IPTG)**

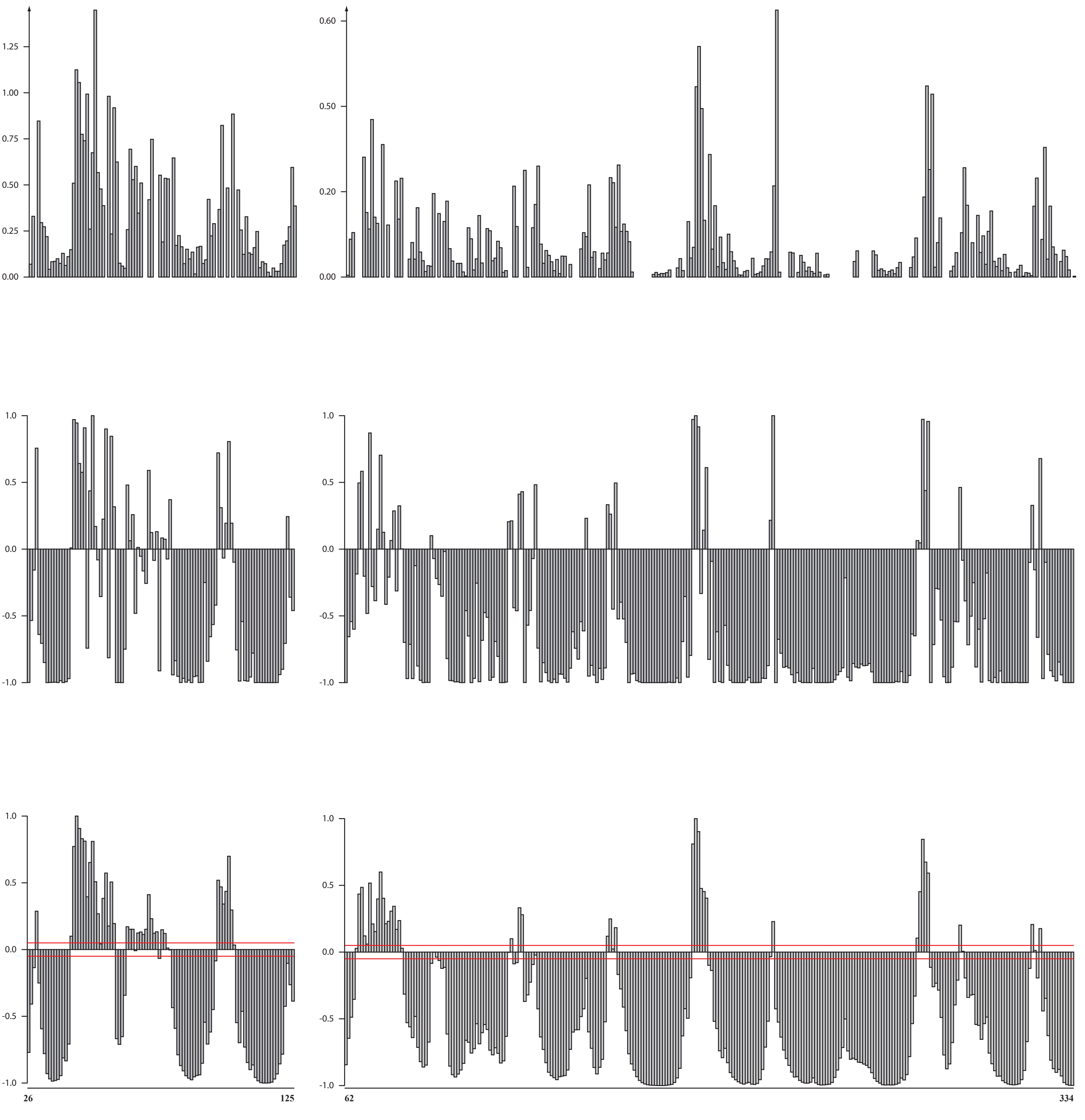

Supplement: Additional file 1 — Figures presenting the chemical shift perturbations and derived confidences as function of the residue sequence for all test cases discussed in the text. Each column corresponds to one test case. Top: Raw CSP data; Middle: Starting confidences; Bottom: Confidences after homogenization. The horizontal red lines displayed on the graphic after homogenization delimit the perturbed (above the upper line), the unperturbed (bellow the lower line) and the intermediate (between the two lines) regions, as determined by SAMPLEX. [file 1471-2105-11-51-S1.PDF]
